# Policy developer’s perceptions on the implementation of National Health Insurance in South Africa: a qualitative study

**DOI:** 10.1186/s40545-023-00564-x

**Published:** 2023-05-05

**Authors:** Vivian Naidoo, Fatima Suleman, Varsha Bangalee

**Affiliations:** 1 Discipline of Pharmaceutical Sciences, University of KwaZulu-Natal, Westville Campus, F-Block, Room F2-519, Durban, South Africa; 2 School of Health Sciences, University of KwaZulu-Natal, Westville Campus, F-Block, Room F2-519, Durban, South Africa

**Keywords:** National Health Insurance, Universal Health Coverage, South Africa, Pharmacist

## Abstract

**Background:**

Universal health coverage has gained significant momentum internationally as the policy solution to address healthcare system deficiencies and promote equitable distribution of quality healthcare. The South African government has adopted this option and developed policy papers for discussion on a National Health Insurance (NHI) system for South Africa. A large part of the policy has been focused on promoting functionality of the primary healthcare system (PHC); to promote an efficient referral pathway. This study sought to explore potential barriers perceived by policy developers that could hinder achieving the NHI goal. Furthermore, given that a large focus is centred on PHC re-engineering, it was imperative to understand participant’s opinions and perspectives on the role of a pharmacist at this level.

**Methods:**

A qualitative research design was adopted in this study. Semi-structured interviews were conducted with ten policy developers that were selected via a referral technique. These were audio recorded using a digital voice recorder on an online platform, transcribed verbatim and saved on Microsoft Word^®^ documents. NVivo^®^, was utilized to facilitate the analysis of data. A thematic analytical approach was used to categorize codes into themes.

**Results:**

The findings revealed that participants were in agreement that healthcare system reform is crucial in promoting equitable distribution of healthcare services in South Africa. However, the reality of this is dependent on addressing key concerns perceived by participants that have been reported as three major themes: (1) the benefit of NHI implementation; (2) concerns about NHI implementation; (3) implications for pharmacy.

**Conclusions:**

South Africa is in the second phase of NHI implementation. This phase is focused on the development of sound NHI legislation and structures. This study identified a number of concerns regarding legislative anomalies and role-player involvement that could compromise the efficient implementation of NHI.

## Background

Universal health coverage has gained significant momentum internationally as a possible solution to address healthcare system deficiencies in low- and middle-income countries [1]. The current South African healthcare system is rooted in the apartheid era that promoted socio-economic and ethnic marginalization [2]. This fragmented system failed to completely address the rising healthcare needs of the country and propagated further inequities. The current system is constructed of two tiers, i.e., the private sector and public sector. The majority of financial and human resources lie within the private sector which services ~ 16% of the population, whereas the public sector services ~ 84% of the population [3, 4]. The country’s healthcare profile faces a quadruple burden of disease, resulting in significant strain on the public healthcare sector. The historical privatization of healthcare created exclusionary structures that made quality healthcare services a commodity available only to those of financial means and disadvantaged the poor, resulting in limited access to quality healthcare services [2, 5]. In an attempt to correct this inequality the South African government has commenced with the implementation of National Health Insurance (NHI) as a health financing mechanism to navigate South Africa towards Universal Health Coverage (UHC) [2].

Universal Health Coverage, advocated by the World Health Organisation (WHO), is centred on removing financial barriers and enhancing access to essential healthcare services [2]. The NHI is a financing mechanism designed to create a single financial pool of funds generated through an increased gross domestic product (GDP) expenditure on health, increased income tax, value added tax and new payroll taxes [6, 7].

During the implementation process, a large focus has been placed on re-engineering primary healthcare (PHC) service delivery by navigating towards a more comprehensive integrated service package. Within the NHI bill there will be contracting units for primary healthcare services (CUPS) that will function within sub-districts to provide necessary services. The CUPS will comprise of clinics, community health centres, network hospitals and private institutions that are contracted and accredited to provide healthcare services under the NHI fund [2, 7]. Thus, there is the need for financial and professional incentivization to motivate role-player involvement and promote service delivery functionality. Furthermore, the objective of PHC re-engineering is to regulate gatekeeping (access and entry into the healthcare system) and support an efficient referral pathway [2]. Therefore, pharmacists are expected to play a crucial role as they are generally viewed as a first point of access to care [8, 9].

South Africa is in the second phase of its NHI implementation (2018–2022). This phase has been largely focused on the development of sound NHI legislation and structures [6]. The primary healthcare system is a central tenet to the foundation of legislative implementation activities, hence this study sought consideration in terms of factors contributing or hindering the efficient functioning of this system. In addition, the study aimed to explore potential barriers that could prevent legislation from attaining the desired outcome [10].

Community pharmacies are likely to pay a key role within NHI. Two thirds of all pharmacies registered with the South African pharmacy council (SAPC) are community pharmacies, and these deliver primary care services, such as chronic disease management, health education and promotion, maternal and child healthcare and immunization in both urban and rural areas. According to a study by Ward et al. the total number of community pharmacies that were registered with the SAPC between 1994 and 2012, increased by 13%. This growth was not at the same pace as the 25% increase in population over the same period. The differences in density between the most rural and least rural provinces decreased from 1.3 community pharmacy per 10 000 residents to 0.72 community pharmacy per 10,000 residents between 1994 and 2012 [11]. Engaging community pharmacies is, therefore, key, and understanding how policymakers perceive how these can be incorporated into NHI roles and be reimbursed is essential.

The objectives of this study were to:

1. Explore policy developers perception on NHI implementation in South Africa.
2. To gain understanding on models of reimbursement of healthcare providers and the PHC service delivery package.
3. To understand policy developers perception on the anticipated role of a pharmacist in the PHC system.

## Methods

### Study design and population

A qualitative study design was adopted in this study with a small sample population. Semi-structured interviews were conducted with ten policy developers that were selected via a referral technique. This approach enabled researchers to engage and gain a deeper understanding and perspective. Policy developers were representatives of both private and governmental bodies that contributed to policies. For the purposes of confidentiality participants practice platform was not reported.

### Ethical clearance

Ethical clearance was granted by the University of KwaZulu-Natal, Biomedical Research Ethics Committee, South Africa (BREC Ref No. BE626/17). Participants were sent formal invitations via electronic mail, with an information sheet outlining the purposes and procedures of the study. Interviews only proceeded once verbal consent was obtained. Participants’ anonymity was maintained throughout the study and a participant identification number was allocated to each participant. Participants were made aware that their participation was voluntary and contact details of all researchers were made available.

### Data collection

Interviews were conducted over a 5-month period via an online platform, due to COVID-19 pandemic restrictions. The use of semi-structured interviews has exhibited significant validity and reliability in conducting qualitative research [12]. The interview questions were designed to explore the perceptions and opinions of policy contributors on NHI implementation. The questions were developed collaboratively by researchers and amended where necessary to ensure that they were concise and clear. The preliminary questionnaire was piloted on eight pharmacists to minimize ambiguity and ensure understanding; and the relevant amendments were made. The interview comprised of 17 open-ended questions that discussed: NHI implementation in South Africa; reimbursement mechanisms; the PHC service delivery package and pharmacist roles and level of readiness. The use of open-ended questions allowed participants to explicitly express their opinions. The use of probing during the interviews enabled researchers to gain a deeper understanding.

### Data processing and analysis

Interviews were conducted in the English medium. Interviews were recorded by a digital recorder and were transcribed verbatim on Microsoft Word^®^ documents. The interviewer remained actively aware of her capacity in questioning to ensure that bias was minimized. Validation of transcriptions of interviews was conducted by the interviewer and one other researcher. Initial analysis was conducted on five transcripts. In this study it was observed that responses were homogenous, and therefore, a discontinuation criterion was established with five additional transcripts. One researcher reviewed all the transcripts and applied codes utilizing NVivo^®^ to co-ordinate the analysis of data. Another researcher reviewed the preliminary codes obtained. Researchers discussed any inconsistencies in coding. The final codes were refined and consolidated into a codebook. A thematic analytical technique was used based on a Braun and Clarke methodological approach to categorize codes [13]. The third researcher then reviewed the coding and any inconsistencies. Verbatim quotations from transcripts are presented in the results section to support the findings.

## Results

### Sample characteristics

Ten policy developers were interviewed from different policy practice settings in the healthcare system. Participants were from various geographical locations across South Africa. From the ten participants, six were male and four were female. The average years of experience of participants was 18 years (Table 1).

**Table 1:** Participant demographics

Participant Pseudonym	Gender	Geographical location	Years of policy contribution experience
Participant 1	Male	Western Cape	21
Participant 2	Female	Gauteng	16
Participant 3	Male	Gauteng	21
Participant 4	Female	Gauteng	11
Participant 5	Female	Western Cape	14
Participant 6	Female	Gauteng	6
Participant 7	Male	Western Cape	12
Participant 8	Male	Gauteng	26
Participant 9	Male	KwaZulu-Natal	20
Participant 10	Male	KwaZulu-Natal	34

The focus of this study was to evaluate the opinions and perceptions of policy developers on NHI. The findings have been reported as three major themes which have been further divided into sub-themes. The three major themes were: (1) the benefit of NHI implementation; (2) concerns about NHI implementation; (3) implications for pharmacy. Tables 2, 3 and 4 present the quantified occurrence of themes and sub-themes from the data.

**Table 2:** Occurrence of themes and sub-themes from transcripts: the benefit of NHI implementation

Theme	Sub-theme	Total number of transcripts	Number of times in transcript
The benefit of NHI implementation	Enhanced access to healthcare services	7	17
	Collaborative practice	6	8

Total number of transcripts: number of transcripts the theme was identified in number of times in transcripts: number of times the theme was mentioned by participant

**Table 3:** Occurrence of themes and sub-themes from transcripts: concerns about NHI implementation

Theme	Sub-theme			Total number of transcripts	Number of times in transcripts
Concerns about NHI implementation	Leadership and governance	Gaps in governmental capacity		10	49
		Overlooking existing pathways		6	14
		Combined public–private platforms		10	25
	Reimbursement	Lack of reimbursement mechanisms		10	25
		Reimbursement mechanisms	Capitation	5	12
			Fee-for-service	4	4
			Outcome-based	6	7
			Hybrid system	4	8
	Role-player engagement	Incentivization to participate		8	19
		Information		7	18

Total number of transcripts: number of transcripts the theme was identified in number of times in transcripts: number of times the theme was mentioned by participant

**Table 4:** Occurrence of themes and sub-themes from transcripts: implications for pharmacy

Theme	Sub-theme	Total number of transcripts	Number of times in transcripts
Implications for pharmacy	Underutilization of pharmacist	10	61
	Training and education	8	27
	The provision of PHC services in a community pharmacy	Refer to Table 5	Refer to Table 5

Total number of transcripts: number of transcripts the theme was identified in number of times in transcripts: number of times the theme was mentioned by participant

## The benefits of NHI implementation

### Enhanced access to healthcare services

Participants were generally optimistic about moving towards universal health coverage by means of the NHI.

“I fully support the intentions of NHI, fully support the principles of NHI.” [Participant 8]

The primary reason for participants being optimistic about NHI, is that NHI will allow citizens equitable access to affordable healthcare services irrespective of their socio-economic circumstance.

“On the positive side, I think NHI is something that has to happen. I do believe that everybody should have equal access to health care. It shouldn’t depend on race, gender, or socio-economic bracket, it should be available to everybody. So that’s the first thing that’s the positive thing about NHI.” [Participant 5].

“At the point of care, patients should not be prevented from getting services because they don’t have funds or they don’t have money. We should not be turning away patients with the structured universal health coverage, and the platform of NHI, which is the funding side. It will allow us to get to that point where a patient can access services without worrying about the fact that they might not have money.” [Participant 10].

### Collaborative practice

Participants were of the view that with the implementation of NHI there will be more collaboration between the public and private sector healthcare professionals which in turn will enhance healthcare coverage.

“I think the mal-distribution between public and private needs to be addressed and therefore we need to be able to design a single system, potentially with an NHI where you contract with public and private, to deliver services to all South Africans and I think that would be a successful formula. And I think that's where the NHI can come in on its own and be able to fill in the gap.” [Participant 14].

Most participants highlighted that the COVID-19 pandemic and vaccination roll-out was an example of how collaboration between sectors could be implemented in the South African healthcare setting. The collaboration between the public and private sector provided a platform for enhanced service delivery as well as access to the COVID-19 vaccine.

“But COVID has also showed us that [collaboration between public and private sector] in the context of universal health coverage or NHI. For example, we were able to use the private sector to do a lot of things when the pandemic started, there was testing done by private sector facilities which was helping government. Now with vaccination with public sector, private sector is coming to assist, then this solidarity fund. So clearly there's room for collaboration between public and private sectors to get involved in providing care, that's the spirit of NHI, organising funding so that we can use both public and private delivery platforms.” [Participant 3].

Similarly, a participant alluded to another public–private collaboration in the form of the centralised chronic medicine dispensing and distribution (CCMDD) programme that has also exhibited the potential for NHI implementation models.

“I mean, here's another example, with the CCMDD programme. We effectively allowed people to collect their medicines from private pharmacies that were contracted under the programme, we just did it. We didn't call it NHI, we just did it. So, you know there are different things that can happen at different phases.” [Participant 1].

### Concerns about NHI implementation

Although most participants were optimistic that NHI may lead to more equitable distribution to quality healthcare services, a few concerns were identified and reported below. Table 3 presents the subthemes for this theme.

### Leadership and governance

#### Gaps in governmental capacity

Participants expressed concern over the South African governments’ ability to efficiently execute the envisioned objectives of the NHI bill. The general perspective was that there is a deficiency in healthcare system planning and management.

“The current proposals in the bill, and the way they plan to structure it, I think, it is all going haywire. I don't think that people have really applied their minds to what needs to be done. We have identified almost four or five constitutional anomalies, and a whole lot of other things that are not aligned to good health structuring of the health system. We don't believe it's necessarily good structuring for health, it's not good health systems strengthening—put it that way.” [Participant 8].

Participants expressed that there is still legislative processes that require attention prior to successfully implementing NHI in South Africa.

“I think this is more a political drive, they're trying to restructure the health system instead of getting the policies right first. So if you don't have policies right you're not going to develop good regulatory mechanisms and legislative mechanisms. Everything is wrong, your structural elements in legislation is going to come up wrong. And I think that's exactly what's happened here.” [Participant 8].

It was further emphasized that there is a pressing need for leadership and strategic direction from experienced and knowledgeable governmental leaders, so that individuals on the ground can meet the desired objectives.

“There is a need to manage and coordinate this entire thing. A need to provide strategic vision, so that those can identify their significant roles and what they need to deliver to meet the strategic objectives.” [Participant 8].

“Hundreds of things were not achieved. So, you know, on a practical implementation point of view on NHI, you know is a really a huge failure today, so we really require kind of need a complete new team.” [Participant 1].

“I worry that there is going to be a lot of task teams that are going to be developed and a lot of debt, but when it comes to implementation and benefit to the patient. Are we really giving the patient, the benefits that they should be?” [Participant 6].

Participants also expressed that without this crucial directive to implement the policy efficiently, the envisioned benefit of NHI to the South African citizen may not be achieved due to the lack of healthcare system planning. In reality participants felt that despite the NHI offering a comprehensive package, without a full complement of resources, efficient implementation may not be attainable.

“You know, the government at the moment, if you ask them the issues, they'll tell you we have a comprehensive pipeline. They're saying we can provide all the services that are required. That's nice! But the reality is that some of these services might not be there in practice, either because of a shortage of staff or certain medicine or equipment, etc. In terms of paper. You know the comprehensive package is quite good. It's a huge list of things, if you look at what is in the white paper, it’s a huge list of things. Well, the trick is now when it comes to the actual implementation. So that’s when you talk about access to services, you're not just talking to access to some list of services, we're talking about access to quality services, meaning that a patient goes to a pharmacy or clinic, and they're able to get the services that they want. So the short answer is that on paper it is comprehensive, which is fine, but in reality when it comes to implementation there is the need for access to quality services.” [Participant 3].

Furthermore, due to the high prevalence of corruption in South African governmental departments, participants were apprehensive about having a nationalized health fund with too much power being extended to the Minister of Health. Participants felt that this fund may be subject to the same mismanagement experienced in the past if not appropriately managed.

“If you really break down NHI, it looks like all the power sits with the minister, not the NHI board. You have to sit and wonder how do you then implement good governance, given the history of what has happened in terms of bad governance and state abuse and the state being taken over kind of thing.” [Participant 8].

“One of the biggest criticisms of the way the NHI has been structured, is that too much power was given to the minister.” [Participant 2].

“Well, the risk of misuse of funds is a big one and it's always there. And it's really at the top, because that’s one of the big issues that we have in the risk engine for NHI is that once you start putting all in one pot.” [Participant 3].

In addition, participant 2 suggested that instead of a single fund a more decentralized financial approach should be implemented in the South African healthcare setting.

“My sense is we need a massive change, I don't think that needs to be a single fund NHI. I think we need to implement some legislation for the private sector that should have been implemented a long time ago. I don't think a national centralised system is going to do it, actually I think the opposite is needed, to decentralise everything, to regional funds. [Participant 2].

#### Overlooking existing pathways

A major concern highlighted in participant interviews is that the NHI bill bypasses the provincial level that exists in the current healthcare system.

“One of the biggest problems is the constitutional implications of NHI, for what's called the competence of national and provincial spheres for health. In essence, the bill kicks the provinces out of health and says NHI will deliver health and national will deliver health. And the provinces will just be contracted to do the work in the same way the private sectors, but they won't be controlling it the way that is currently set out in the National Health Act.” [Participant 9].

Participants felt that introducing an entirely new healthcare system structure instead of building on the current framework may be more difficult to implement and achieve. This is due to the fact that the NHI bill does not take into consideration the macro and micro factors experienced at a provincial level.

“I have some questions about what's sometimes called the path dependency with respect to the model that South Africa’s selected. So for example, in Australia and Canada. The model of NHI is provincial as a state run solution, and in countries like, Netherlands and Germany, an NHI model is decentralised to multiple schemes, multiple funds, but like our medical aids. Now both of those two routes would be more in keeping with the principle of path dependency, and what path dependency talks to is that you build in institutions and the framework you have, whereas our NHI proposals are very much kind of starting afresh as if we had nothing. It's kind of lets build this mega national fund that replaces everything else. So, you know, I think that makes the South African proposal a more difficult option to achieve. The issues of the locations of powers between National and the provinces are extremely extraordinarily complicated.” [Participant 1].

Participants also expressed that the formation of CUPS does not take into account the provincial constitutional implications to deliver care and that the introduction of parallel structures for the provision of care may be counter-productive.

“You know, we have done what is in the National Health Act for the last 17 years and now we want to create separate parallel structures that totally then ignore the provinces. And now we want to create these other structures that potentially national can control. Yes, in reality, each clinic and health service belongs to a province. So even if you contract with that CUPS, how does that CUPS then contract with the individual province? You can't ignore the provinces in this equation. So why are you creating middle link structures, when all you need to do is contract directly with the provinces. So it seems to be a deliberate attempt to undermine provincial constitutional mandate to deliver care and deliver health.” [Participant 8].

#### Combined public–private platforms

Participants expressed that the current healthcare system limits access to healthcare services and is based on an individual’s socio-economic standing. A majority of participants highlighted that to overcome this barrier and achieve the objectives of NHI, the government will have to introduce a combined public–private platform that enables the public and private sector to provide healthcare services to a patient within the fund.

“I mean from the treasury perspective, something that we feel is a really important feature of National Health Insurance is a mixed provision platform both public and private.” [Participant 1].

“The multiskilled clinic that they talk about at the primary care level, those kind of ideas are great. The teamwork approach to care is perfect. If you look at the resources in the country. You have 20% of the population in the private sector, 80% in the public sector, but you have over 50% of GPs in the private sector, 50% in the public sector. You got almost 70% or 80% of the pharmacists and dentists in the private sector and a small amount in the public sector. So, any notion that government might be able to run NHI on their own is absolutely wrong, if you want to be successful and you want to work towards a successful system, you need to have two sectors- a private and a public, you need to work a relationship where they all work together as providers of service in a single health system. That's what you want to work towards in your structuring.” [Participant 8].

Participants felt that introduction of the combined public–private platform may be vital in enhancing accessibility to necessary healthcare services.

“Part of helping to solve the country's problems and helping people, the biggest reform that's needed is to really make the kind of potential for provision options much more equitable and accessible, so people can just use what is close to them.” [Participant 1].

One participant expressed that the efficiency within the private sector may be more cost efficient to the government should a combined public–private platform be introduced.

“I think one of the important things that the government needs to understand is that in the private sector, we run incredibly efficiently because it is financially based; you cannot run an operation that is not financially sound. And so I truly believe that in the private sector we can offer these services at a cost efficient basis for the government, and they actually may find that it is cheaper than in their own public sector facilities.” [Participant 5].

However, it was observed by researchers that although this combined public–private platform is necessary, there is concern regarding how this platform could be standardized when majority of resources are within the private sector, as opposed to an under-resources public sector.

“I think it's actually going to slow things down a bit. Currently, those that are on medical aid is about 16%. You know, that gap between 16% and 84% is where the problem is, because the resources are within 16%, and so therefore that 16%, needs to deliver NHI successfully. I don't see how it's going to work.” [Participant 6].

Within a combined public–private system, participants raised further concerns regarding the understaffing in public healthcare facilities that has placed the system under significant strain.

“The thing to bear in mind is that the disparity between private and public in terms of staffing is still a huge concern, we probably have about 40–50% of health, a staff serving 20% of the population and the rest serving the other 80%. So the clinics and primary hospitals are under extreme pressure. It is relentless pressure to work there.” [Participant 7].

A major concern highlighted is that the combined public–private platform may be subjected to individual interests that promote personal gain.

“Mistrust is the one thing and then the other is commercial interest from the private partners side. I can come to the government and say, I have this really great platform that can help you. But, what I actually try to do is generate revenue or I want to get a tender, or my interest is actually I want to make money. It's not really, I want to help, it's actually that you need to take me on so that I could generate revenue. And so I as a person have a problem with commercial interest in healthcare, which I do understand the principle of making a living. But in some cases, there is really undesirable high remunerations in the end at the cost of the patient.” [Participant 4].

Another participant raised concern that the introduction of a combined public–private platform may only be beneficial in urban areas that are populated with these private sector facilities and not necessarily reach the desired communities in rural areas.

“I'm unsure how that is going to filter down to the people in the remote areas. So I find that the public private partnership is going to work in the urban areas, where there was access, already, but you know we're just improving it, but where people really need this in those geographical areas you know like your deep rural areas, are we going to reach them with these public private partnerships? I'm just worried that it's a big advantage to people who already have.” [Participant 6].

Conversely, another participant stated that the provision of these contracting services within the combined public–private platform may incentivize healthcare providers such as pharmacies to move in to these low coverage areas.

“So I said earlier, this would be a good way of securing income, Because the whole idea of accreditation would also mean that it will force retail pharmacies for example to go to areas where they are needed, five retail pharmacies within a small radius? They'll only accredit people in strategic areas to make sure that there’s coverage. So, places that don't have retail pharmacies, for example, would end up having because of the accreditation system.” [Participant 3].

### Reimbursement mechanisms

#### Lack of reimbursement mechanisms

A common concern expressed by participants is the lack of information on reimbursement mechanisms for healthcare providers expected to render services within NHI.

“This is not detailed in the legislation at all. What is it actually going to mean for you on the ground. And ultimately, people are worried about the quality of the working environment, they are worried about the reimbursement at the end of the day.” [Participant 2].

It was observed that participants felt that engagement about reimbursement with healthcare providers should be prioritized to ensure that the system functions efficiently.

“Public–private engagements can be used to ensure that things work very well, so that they can always make a case for themselves.” [Participant 3]

Participants expressed that without this engagement healthcare providers may be less likely to participate in the envisioned activities under NHI.

“The Department of Health may come out one day with some poorly thought out reimbursement proposal and the professionals just reject it the next day, that can happen. You need to be able to construct something that's mutually beneficial and works for the country.” [Participant 1].

“I hope that they're not going to introduce it and say look, this is the fee and you guys go ahead and make it work yourselves, because I think that would be a recipe for a bit of a disaster. You know the fee will probably be too low for what's actually required and they will end up under servicing.” [Participant 2].

One participant emphasized the need for health information systems analysis after NHI policy implementation. This is to determine if the system is operating efficiently with suitable reimbursement.

“You can't remunerate and not bother to analyse the engagements and what happened. So, a critical success factor would be the presence or absence of some kind of health information recording system. Whether the switching type, information exchange, or whether you'd have an electronic health record, that everyone then captures information on.” [Participant 8].

It was evident that majority of the participants felt that the current reimbursement mechanism is not suitable and new mechanisms need to be introduced with NHI implementation.

“It certainly cannot work in the current framework, the way we get paid at the moment, we need to remove that from the system, and come up with a new model of intervention.” [Participant 10].

Furthermore, participants expressed that currently in the private sector, medical aid payment coverage particularly in a pharmacy environment is problematic, as pharmacists interventions are not recognized or reimbursed.

“We do have those fees we're allowed to charge in private sector, but very few of the schemes, if any are willing to pay for those most of the time.” [Participant 2]

“If someone says please offer this, people will say but the medical aid is not paying. A lot of pharmacist feel that if the medical aid is not going to pay, I’m not going to be able to do that.” [Participant 4]

Furthermore, another participant expressed that the use of the single exit price and dispensing fee used in the private sector pharmacy environment has hampered professional progression due to the lack of reimbursement mechanisms.

“Because of the single exit price and the dispensing fee, and especially in the hospital sector, because of the decision by the hospitals, not to charge the dispensing fee but only to sell at the SEP. That has resulted in pharmacists having to tailor their offerings to what is reimbursed. And I think it's hampered the professional development of both community and hospital pharmacy when compared to other parts of the world. And it's really that point that reimbursement drives quality of pharmaceutical services and the scale and the extent of those pharmaceutical services. I think that NHI at least in theory, offers a way out of that conundrum.” [Participant 9].

#### Primary healthcare reimbursement mechanisms

Taking into consideration that this study is focused on the PHC environment, participants were further probed to give their opinions and perspectives on the different reimbursement mechanisms that could be utilized in a PHC setting. Four prevalent mechanisms that were discussed are presented below.

##### Capitation

Participants referred to the NHI bill adopting a capitation model for reimbursement that will be introduced as a service provider package for a specific population.

“But in the basic system, the basic form of reimbursement is capitation, particularly for the core PHC team.” [Participant 1]

“So firstly, the NHI envisages that it will determine the package prices for the whole package and all of the elements of a package. There will be a price setting function within the NHI and then reimbursement for those services. So it's not aimed at building a system, it's aimed at extracting as much value as it can out of the system. In other words, it won't be a price taker, it will be a price setter. The way in which it's written hints at a capitation system. But it hints as a capitation system for a group of patients registered with a particular set up, which includes all of the primary care services.” [Participant 9].

However, it was noted that some participants felt that this model may not be suitable in the South African healthcare setting and implementers should give it careful consideration.

“The current draft bill talks about capitation for primary healthcare, again it's this very sort of a loose concept that I don't think the drafters of the bill have really given much thought to how that's going to be implemented given the fragmentation that currently exists. So capitation ideally you want to be paying an amount per month, per patient to a team that's going to be handling. But given that we don't, we don't have a proper benefits, we don't know how it will be delivered. We don't even have the existing data for the current system at the moment to be able to estimate what decent capitation fee would be in that system.” [Participant 2].

Another participant preferred the capitation model and felt that it promoted continuity of care between healthcare providers and patients.

“The main reason why I like capitation and most people like capitation is because of this whole principle of continuity of care in primary care. So you know, you and your family have your doctor, your health team or pharmacists that you go to, and you develop this relationship with them.” [Participant 1].

The same participant further elaborated that although the capitation model supports continuity of care, it may not be suitable in a community pharmacy environment.

“I don't know much about pharmacy reimbursement in NHI systems but if you had continuity of care, we know the person would typically go to the doctor and then they would go to the pharmacist and get their medicines. Then I guess you could bill them some form of capitation payment. But if that doesn't work very well in the pharmacy environment and people tend to shop around, one week to one pharmacy, another week to another pharmacy. So it depends on how important this principle of continuity of care is for pharmacy. It might be that pharmacist themselves haven’t actually quite thought through this concept of continuity of care.” [Participant 1].

In addition, another participant felt that a per capita amount does not consider the population demographics for the provision of services.

“There are so many questions around this, because it would need to take into account the disease profile of the community. It will have to take into account the age groups, or the mix of the age groups and all the different demographics, it will have to take into account all of the factors. It becomes rather difficult to use that model.” [Participant 7].

“Because if you look at geographically we have different burden of disease in the different areas. And yes, in rural areas you have more population and few practitioners, and in suburban environments you have too many practitioners and maybe too few patients. And obviously, these differences are rare, so you can't be remunerating everyone the same rates, but you can structure a kind of a risk based reimbursement structure based on the prevailing risk that's allocated to practices.” [Participant 8].

##### Fee-for-service

Participants expressed that the current fee-for-service model is preferred as they are reimbursed for the services offered.

“I know that they want to move away from the fee-for-service, which is what we're comfortable with. We do x, we get paid for x. Now, suddenly we going to have to do, I don't know how many services and we get paid a fixed amount.” [Participant 5].

Whereas another participant expressed that it may be beneficial to introduce a fee-for-service model of reimbursement as a foundation to build other reimbursement models.

“What I'm hoping is going to happen first is that we'll start with a fee-for-service type of offering in primary. Where they can start to kind of see through the pilots what the population actually cost in that area on a fee-for-service basis so that we can translate that to some kind of capitation payments from a primary health care level.” [Participant 2].

Another participant elaborated that in a pharmacy setting a fee-for-service model may be better suited for services render.

“My understanding is that there are two models, which is capitation, and the fee-for-service. If we go for the fee-for-service, maybe you can go to the pharmacy, get your medicines dispensed. Then review a blood pressure patient for example. So get paid for everything that is offered, a fee for every service.” [Participant 4].

It was, however, suggested that the fee-for-service model left room for fraudulent activities.

“I just feel that the system currently… this fee-for-service, doesn't work. It doesn't work! The list of people providing fraudulent claims!” [Participant 6]

##### Outcome-based reimbursement

Many participants alluded to adopting an outcome-based reimbursement model that may foster a sense of accountability among healthcare professionals rendering services.

“I'm very biased towards value based outcomes. I believe that even doctors should be paid to some extent based on the outcomes. So I think that if we start reimbursing people based on outcomes, people will take more responsibility. For example, if we're actually going to say, we're going to reimburse based on the number of asthmatics, are you offering asthma pharmaceutical care? Is there decreased emergency visits? And then depending on that, then they get something.” [Participant 6].

“NHI model needs to be redesigned or re-engineered. Outside of time, measuring outcomes. So were you able to achieve an outcome in that patient? In other words, if you are able to keep the patient out of the emergency room.” [Participant 10].

Another participant further elaborated that this model allows for each patient to have an individualistic treatment experience based on their health needs.

“Firstly, patients are not mass produced. So your needs and my needs, although we are both females is not the same. We can be born on the same day, we can have the same features. We can even talk the same language, but, our needs are not the same. So a system should cater for unique needs.” [Participant 4].

In addition, it was highlighted that using a more outcome-based reimbursement model can reduce the potential for over-servicing that is seen with the fee-for-service model*.*

“They talking about having risk based payment models, reimbursement models. And of course we've never had that in the private sector, we've always been fee based, fee-for-service. And we also need to change because fee-for-service can lead to over servicing.” [Participant 5].

##### Hybrid system

Some participants felt that a hybrid system should be adopted to reimburse healthcare professionals.

“I potentially see a hybrid system. Where you get paid a salary and then potentially a different mechanism based on load and things like that. So it will definitely be a risk based remuneration system and potentially in primary care, then there will be definitely a form of primary care capitation system. Then linked to the capitation system they will still have to be kind of line by line, exchange of data monitoring on the type of services delivered and services the patients access, and it will also help in assessing the extent of the quality of care delivery.” [Participant 8].

“Like a mix of reimbursement mechanisms that it would be based on capitation but then a little bit of fee-for-service, and a little bit of performance incentives.” [Participant 1]

### Role-player engagement

An additional theme identified was surrounding key role-player involvement in NHI activities. Key role-players are individuals from various organisations, interest groups and departments in the healthcare system that are involved in the implementation and maintenance of NHI [14]. The findings are further elaborated in the below sub-themes.

#### Incentivized participation

It was observed that participants felt that key role-players might require further incentivization to participate in NHI activities. This incentivization is not limited to monetary benefit but also to encourage healthcare providers to get involved.

“You know it's got to have appropriate incentive arrangement but also this is not just about money, it's also to do with a sense of being part of a bigger picture and part of helping to solve the country's problems and helping people. You know you can design centres which encourage private providers to be part of the publicly funded system.” [Participant 1].

Similarly, another participant felt that a more holistic approach is essential in encouraging role-player buy-in. This could ensure that providers are profitable yet still achieve the policy objectives.

“They will also need to factor in that there has to be a return on investment. We can't be doing it at cost neutral. You know that's not how anything works. We need to be able to live and to prosper. So it's a balance of looking after patients well and also keeping the whole engine of the healthcare industry running.” [Participant 5].

However, it was clear that the monetary avenues remain the primary form to encourage role-players to participate.

“So coming from a finance side, we tend to find that people respond to tariffs a lot. The design of tariffs and the level of fees. So if you incentivize people enough they tend to respond to the tariff.” [Participant 1].

#### Information

A sub-theme identified was surrounding the lack of information and communication regarding implementation processes of NHI and role-player involvement and roles.

“I think the communication from the Department of Health has been quite bad. And unnecessarily raised the alarm as well. I think that they haven’t really succeeded in bringing the professionals along with in many cases.” [Participant 1].

“We don't know those details yet, so you can't blame the health professionals for being sort of hesitant to engage with all these things when details for them on the ground, are not kind of spelt out yet. So we're being asked to support National Health Insurance but you don't know from a practitioners point of view, what that's going to mean for you.” [Participant 2].

Participants expressed concerns that there is a lack of information surrounding NHI activities. Therefore, without the appropriate dissemination of information the objectives of NHI may not be achieved.

“That is the anchor points of the entire health system reform success. All this health system strengthening tools that you want to implement, reforms, etc., will only be successful to the extent that we understand. Without information, we are blind. And therefore, our important strategic objective is to sort out that information source.” [Participant 8].

Furthermore, it was noted that participants felt that in general healthcare professionals were not informed on the NHI and the progression of implementation.

“The average healthcare professional is not informed! Either it is not really caring about the process or on the other hand have so much trust in that it will be handled on our behalf. In terms of detail and being informed, I don’t think the average healthcare professional is.” [Participant 4].

However, it was noted that there was a difference in opinion as some participants did feel that information has been disseminated to stakeholders and engagements have taken place.

“I think if you are a stakeholder, you have been engaged. I think you should be quite well informed. I mean the government has put out a couple of white papers, we have answered those we have sat with the government.” [Participant 5].

“I think if they're not well informed, then they really shouldn't be in the health space, that is my opinion. Because if you think about the last 10 years, we are talking about NHI and universal health coverage. There's so much of literature, data, information and information sessions.” [Participant 10].

## Implications for pharmacists

Given that a large focus of this study is centred on PHC re-engineering and the proposed integral role of a pharmacist; it was imperative to understand participants opinions and perspectives on the role of a pharmacist within NHI implementation. The findings are reported in Table 4.

### Underutilization of pharmacists

Participants felt that pharmacists are underutilized healthcare professionals and the current structure of the healthcare system allows for very little inclusion in healthcare provider teams.

“Firstly, pharmacists are very underutilised as far as abilities and professional scope of practice.” [Participant 5].

“The role of the pharmacist is very limited in comparison to what they could be doing at the moment. Depending on the environment that you're in, it's a very kind of get the patient through the system, out the door kind of thing. There is not a lot of focus on medicines counselling, medicines review, questioning the patient about existing treatments etc. You know it's unfortunate and it's not because of pharmacists, it's the environment that they work in that incentivizes that.” [Participant 2].

Participants expressed that the reposition of pharmacists as PHC providers will enhance service delivery and improve functionality of the referral pathway.

“I always think pharmacists can do more. The Pharmacy Council says you can't do this, you can’t do that, but you're trained to do it. For me that's really a lot of missed opportunities. When I go and see a pharmacist and they say I know what is required but let me go talk to the doctor and get a prescription and then you can come, I will help you. Don’t you think that is a waste of time. When the pharmacist can simply help me. So there is a need to look at the scope of practice so that we can improve coverage in some cases and avoid these situations. In any case, I then have to go and pay for the doctor. Currently, because I don’t have insurance, so I paid for the doctor and then I go to the pharmacy, then I pay again. So there is scope to enhance what the pharmacist does. And I think in the context of NHI that would be very helpful.” [Participant 3].

“I think the PHC package is fairly comprehensive; what I do think needs to happen is that there is a lot more that pharmacists can contribute to that package. So when you look at that package, it is very GP oriented. And I believe there's a lot more than pharmacists could do.” [Participant 5].

A common opinion expressed is that the role of a pharmacist has been minimized to pill dispensers and further limited by other administrative duties [8].

“They're nothing more than pill dispensers broadly speaking.” [Participant 8]

“Unfortunately, from my perspective it’s kind of just becoming dishing out medicines and there's a lot more that pharmacists could be doing. They're just not paid for those services.” [Participant 2].

“I think pharmacists working in South Africa, especially in the retail setting are kind of seen or perceived to be store keepers. So they don't really have a defined role; there is very limited pharmaceutical care that is currently being provided by our pharmacists. It's really about meeting targets and getting the queue to be shorter.” [Participant 6].

It was also expressed that pharmacists have adopted a gatekeeper role that this role is not recognized by other healthcare professionals.

“I think pharmacists are really keepers when it comes to medicines. Unfortunately, I think with the way our healthcare system is structured, that they are not seen like that and yet trained to be that. Unfortunately, in my experience, I find that our system, the multidisciplinary team does not always involve pharmacists. So at the end of the day, most pharmacists knowledge is just shelfed somewhere, which is quite unfortunate.” [Participant 6].

Many participants expressed that pharmacists should take on a more clinical role in the healthcare system as opposed to the product centred role currently adopted.

“So I think there is a need for more focus on patient outcomes and clinical outcomes rather than focus on the drug and the side effects.” [Participant 10]

“Unfortunately, pharmacists at the moment are very product based and the services are medicine based. And I would like to see this move into clinical practice healthcare services.” [Participant 5].

It was also further highlighted that pharmacists are integral in enhancing access to care as they are well-positioned within communities. This will be beneficial in also reinforcing the referral system.

“I believe community pharmacists, are one of the most accessible health care professionals within a community; and trusted healthcare professionals that has a huge role to play in NHI.” [Participant 5].

“I think this is exceptionally important. Firstly, because that is your point of action into the healthcare system. Secondly, how many patients come in with “I’m not feeling well”, “I have a headache”, “look at this rash”, "I'm having something here," or whatever. A lot of patients can be helped, pharmacy initiated therapy! So, primary healthcare is not only your first point of access, majority of all of your minor ailments is treated in a primary health setting. And primary healthcare settings it's also ideal to prepare a patient and the system for transferring a patient into secondary healthcare, because we don't want to end up with a patient with simple flu or sinusitis, to walk into a tertiary hospital demanding attention from a specialist. That is a waste of resources. So primary healthcare is exceptionally important and that is ideally where a pharmacist is placed.”[Participant 4].

Many participants expressed that pharmacists are well-positioned to ensure continuity of care and promote good medicine taking practices and adherence.

“The pharmacy is also ideally placed to do the continuation of care, which is largely neglected.” [Participant 7].

“I think the pharmacist has a key role with the continuation of treatment once a patient is diagnosed and put into primary care. I think with NHI, that pharmacists will play a much more prominent role.” [Participant 10].

### Training and education

Majority of participants felt that pharmacists are underutilized and further inclusion is required with NHI implementation. However, it was observed that participants felt that further training and education is required for pharmacists to take on these newly envisioned roles.

“Well, I think there’s further education and training that needs to be done here.” [Participant 3]

“The criticism is, I think we're going in the right direction based on the country's needs, but I think you actually need to really looked at the system properly because the needs are not in the places where you potentially will be directing your efforts and component is you haven't structured the pharmacy curriculum for this new scope of practice correctly. To me, you need to lump it all together as part of a full blown primary care service training system, and then you are qualified as a primary care practising clinician and pharmacists at the same time.” [Participant 8].

A primary reason why participants felt that further training is required, is their view that the current product centred role allows for very little clinical-based involvement. Therefore, further training will ensure that pharmacists are competent and confident to undertake these anticipated roles.

“You know, since training, you haven't had much of an opportunity to do that kind of work in a community pharmacy, that's been the reality. So, you need to do some CPD activities, more theoretical CPD activities and then some on the job activities. Qualifying things, just to enable the pharmacists to have that confidence to do it. I mean it could be well within the scope of pharmacists, they just haven’t really had an opportunity to practice that kind of thing, so additional training is needed.” [Participant 2].

### The provision of PHC services in a community pharmacy

Participants were further probed on services that should be rendered in a community pharmacist in which a pharmacist is reimbursed as a PHC provider under NHI. The most prevalent services mentioned are reported in Table 5.

**Table 5:** Types of services to be rendered in a community pharmacy as expressed by participants

Service	Total number of transcripts	Number of times in transcripts
Immunization	7	12
Disease management services	6	12
Screening and monitoring services	6	9
Primary Care Drug Therapy pharmacist (PCDT)	5	6
Pharmacist Initiated Management of Antiretroviral Treatment (PIMART)	4	7
Antibiotic stewardship	3	3
Selective family planning	3	4
Treatment and diagnosis of minor ailments	3	4

Total number of transcripts: number of transcripts the theme was identified in number of times in transcripts: number of times the theme was mentioned by participant

A majority of participants highlighted that immunization services should be included in the service delivery package of a pharmacist at a community pharmacy level under NHI.

“So, our pharmacists could render either family planning, immunisation for kids, immunisation for adults, including flu, COVID, your travelling immunizations.” [Participant 7]

Furthermore, participants expressed that there is a need for a standardised provision package for screening and monitoring services that enable a pharmacist to also provide the necessary disease management services and ensure continuation of care.

The pharmacy will render continuation of care service at a fixed cost. So that continuation of cases could include your regular health checks, such as blood pressure, blood glucose, cholesterol, it would include advice as to the medicine they are using regarding how to manage side effects, when to go back to the doctor. The patient could find this continuation of care model, extremely valuable, because at the clinic, they will see a doctor or a nurse, once in six months. So on a monthly or bimonthly date, the pharmacist will do these kinds of health checks, provide advice and counselling.” [Participant 7].

A few participants also felt that the introduction of Primary Care Drug Therapy (PCDT) services at a community pharmacy level would enhance the scope of practice of a pharmacist. In addition, based on the unique knowledge and training of a pharmacist on medicines, they may be well-positioned to ensure the safe and rational use of medicines.

“I'm not sure if you know about primary care drug therapy. So PCDT, that's a great service! I’m part of the training of PCDT pharmacists and there’s such a lot of pharmacists that complete the training but for some reason they never go on and get the permit and actually practice. But that gives a pharmacist a broader scope of practice.” [Participant 4].

“I think, pharmacists, being a bit more aware for example of, multi drug resistance with antibiotics, they might actually do a better job. They probably wouldn't prescribe as many antibiotics as doctors prescribing at the moment.” [Participant 2].

Some participants felt that the inclusion of pharmacist initiated management of antiretroviral treatment and HIV pre- and post-exposure prophylaxis services is crucial to combat the prevalence of HIV and AIDS in South Africa.

“Other issues, where the country is struggling with not changing outcomes in certain areas and one critical area in South Africa is the HIV and AIDS environment. I think if you look at the PIMART pharmacist now.” [Participant 10].

“We've started training pharmacists on pharmacist initiated management of ART. If we look at the quadruple burden of disease, HIV is a big one. And I really do believe that pharmacists are seeing these hard to reach populations that are not going to hospitals and doctors. So they could assist with PEP, PrEP and first line ARV's.” [Participant 5].

## Discussion

The efficient and effective execution of a policy is dependent on understanding the overall aim the policy was designed for [10]. To meet these aims, proper planning and deliberation is essential. Therefore, to ensure that the aims of NHI are achieved, it was vital to obtain perspectives from key policy developers in the South African healthcare system in hope that their feedback results in a more effective implementation strategy.

The aim of NHI is to attain UHC in the South African healthcare system. The general sense was that participants supported this transition, with the primary reason being equitable access to healthcare services irrespective of an individual’s socio-economic factors. The NHI was designed to pool funds to achieve the progressive reality of the right to accessible quality healthcare services. The policy shift intends to minimize the financial barriers to healthcare and address the inequalities inherited from the past [1]. Many participants expressed that the reality of UHC implementation in the South African healthcare system could be seen in the current COVID-19 vaccination rollout that enabled citizens to utilize both the private and public sectors. Furthermore, there have been international examples of countries that have commenced with UHC implementation, such as Rwanda, Indonesia and Ghana; and have made significant progress in increasing their healthcare service coverage [1, 15, 16]. With that said, there were concerns that a single-fund approach may not provide the intended results and equitable distribution of healthcare in South Africa and UHC could likely remain an empty promise unless these concerns are addressed.

One of the key concerns was that the South African government lacked the ability to implement and manage the objectives of NHI. Successful healthcare system planning and management should address the economic, community and social determinants of health. Efficient planning is crucial in ensuring that the policy meets the intended need and that the healthcare system remains sustainable and adaptable as it navigates changes [17]. Participants expressed that there was a deficiency in healthcare system planning that may result in ineffective system structuring. The fear is that the aims of NHI may be lost due to this misalignment. There is merit to this concern as low–middle income countries are usually burdened with resource deficiencies, complex population socio-economic factors and diverse disease patterns in comparison to developed countries [18]. International examples of the resulting effects of poor healthcare planning and co-ordination can be seen in countries, such as Haiti, Somalia and Afghanistan; that resulted in inferior population health profiles and inadequate healthcare system infrastructure to support their endeavour to build and improve[19]. Participants also expressed that there is a pressing need to revisit the deficiencies in current healthcare policies and develop appropriate legislative and regulatory mechanisms before implementing newer policies.

Participants also felt that there is a need for strategic vision, leadership and strategic direction to plan, implement and manage the complexities within the South African healthcare system. Participants felt that this lack is evident by the stagnant growth and progress since commencing with the NHI implementation process. Vision and leadership is a crucial concept of the strategic planning process. Appropriate strategic vision and leadership facilitates organisational direction and the ability to construct robust strategies and achieve the vision [20].

Corruption was a major concern that was echoed by majority of participants. The consensus was that by centralizing a health fund without appropriate oversight may result in it being subject to the same mismanagement and corruption historically seen in South Africa [21]. Good governance is a crucial factor to ensuring that national healthcare systems operate optimally. The effects of corruption may not present itself in the immediacy of policy implementation, but later, resulting in slow corrosion of the system. Research has linked 1.6% of annual deaths globally in children under 5 years to corruption in the healthcare system [22]. In the Philippines, the effects of corruption resulted in decreased patient satisfaction, longer patient waiting times and reduced access to healthcare services [23]. Tanzania, Uganda and Ukraine are other countries that have acknowledged that corruption is a major barrier that they experience in providing quality healthcare services [24]. Therefore, implementers need to ensure that appropriate governance and regulatory mechanisms are introduced and enforced.

Another, noteworthy concern is the issue of creating parallel pathways. A few participants felt that implementers have ignored the provincial level of the current healthcare system and have not carefully considered the complexity of issues experienced at local levels of care. The effective implementation of healthcare policies are not limited to one entity, but is a reflection of the collective efforts of national, provincial and local governments as well as private institutions. Therefore, the responsibility for appropriate healthcare system management is shared [17]. This strategy for development should take into consideration the different values and perspectives that have to be integrated within the structure [8, 25]. Similarly, a study based on the district healthcare system in South Africa found that there is a pressing need to bridge the health inequities at a district health level prior to NHI implementation [1]. This reinforces the opinion that implementers need to look at existing structures and care pathways in the South African healthcare system and evaluate how past and present factors within the system may influence the successful implementation of NHI. An international example from a study conducted in Egypt found that health policies and the overall institutional setting of the healthcare system limited Egypt’s endeavours to implement UHC (25).

Another integral aspect of NHI implementation is dependent on private healthcare provider involvement and sharing the responsibilities to achieve equitable healthcare in South Africa. Majority of participants supported the idea that private healthcare providers should render services under the fund to enhance healthcare coverage. This combined public–private platform enables citizens to access healthcare across private and public healthcare facilities. However, concerns were raised that due to the disparity created from historical events, extensive work is required to standardize the public healthcare sector to ensure equitable access to quality healthcare services. With that said, regulatory mechanisms are also required to ensure that private healthcare providers do not use this platform to promote their personal interest and that the availability of services are purely to meet the healthcare needs of the population. This also includes an ethically sound contracting and accreditation process that encourages private healthcare providers to move into areas that lack healthcare coverage.

A recurring theme seen in this study is the concern of lack of reimbursement mechanisms for private healthcare providers [8]. Participants felt that there is insufficient information on the reimbursement processes within NHI. Similarly, in other studies conducted by researchers, a common theme documented is the concern that there is a lack of information on reimbursement mechanisms for healthcare providers expected to render services within NHI [8]. It is also important to note that participants had mixed feelings about the potential mechanisms of reimbursement, i.e., capitation, fee-for-service, outcome-based and a hybrid system. However, the general sense was that participants preferred an outcome-based reimbursement approach as it fostered a sense of accountability from healthcare providers by ensuring positive health outcomes. Therefore, consultations with private healthcare providers needs to be prioritised to find a suitable mechanism of reimbursement to promote participation.

The need for role-player engagement was another theme that was identified. Participants felt that involvement and inclusion of key role-players within the NHI implement process is essential in promoting incentivization to participate and ensure that the objectives of NHI are attained. In additions to that, participants felt that the South African Department of Health needs to relook at their mechanism of communication and the avenues that information is disseminated. Participants expressed that they felt that information and communication on NHI implementation should be more detailed and promote inclusivity. Similar challenges were also observed in a study on Malawi; which found that meaningful stakeholder engagement and communication was essential in aligning health policy activities and promote inclusivity [26]. With that said, this responsibility is also shared; healthcare providers need to ensure that they engage and remain up-to-date to ensure that they are equipped to transition into these roles. Internationally, governments and researchers have recognised the impact of communication as an instrumental tool for policymaking. The effective implementation of this tool can enable a two-way dialogue with the public and stakeholders and reinforce transparency and accountability [27].

Given that a large focus has been on PHC re-engineering, researchers felt that it was imperative to gain perspectives from policy developers on the role of the pharmacist in this setting. It was evident that a large majority of participants felt that pharmacists are extremely underutilized and there is room for them to take on more responsibilities as valued healthcare providers. Some participants felt that a pharmacist service package should work in cohesion with that of a general practitioner. This will enable pharmacists to provide and prescribe within a standardized service package and refer to a general practitioner when required. Similar to other studies, participants felt that the role of a pharmacist was burdened by administrative and pill dispensing duties, with little clinical focus [8]. However, it was highlighted that a pharmacist is seen to be a gatekeeper to the healthcare system, and therefore, it is beneficial for a pharmacist to adopt a more clinical role.

It was also noted that participants felt that pharmacists, being gatekeepers, are well-situated to implement health interventions and promote access to essential healthcare services. Consideration should be given to ensure a standardized service provision package. Furthermore, participants felt that services such as immunization, disease management, screening and monitoring and PCDT services should be included in the pharmacist service package. Participants felt that with the unique skillset and knowledge of a pharmacist that PCDT services and disease management will promote better health outcomes. It was also expressed pharmacists are easily accessible and the concept of continuation of care and an enhanced referral system will be strengthened. Furthermore, it is evident that the inclusion of a pharmacist in the PHC service package is crucial; there is still the need for engagement between cadres of healthcare professionals to ensure that each role and scope is clearly defined for an efficient service delivery platform.

### Study limitations

The findings of this study is based on the interviews with policymakers and may not reflect the viewpoints of all stakeholders in the South African healthcare system. It would be beneficial to repeat this study among other stakeholders in the healthcare system to corroborate and expand on the findings.

## Conclusion

The implementation of NHI could address the fragmentation and inequalities that exist in the South African healthcare system. However, to ensure that the objectives of NHI are achieved, conscious efforts by policy implementers are required to address crucial deficiencies in planning and execution processes. Planning, monitoring, documentation and measurement of health needs and outcomes are vital in the healthcare policy design to determine or measure the impact of the policy [17]. This study identified noteworthy concerns that could compromise the efficient functionality of NHI and attaining UHC. Therefore, proactive and positive engagement with key role-players is essential in interrogating and resolving the inadequacies that arise with NHI implementation.

## Data Availability

The data sets generated or analyzed during this study are included in this published article.

## Abbreviations

CCMDD: Centralised chronic medicine dispensing and distribution
CUPS: Contracting units for primary healthcare services
PCDT: Primary care drug therapy
PIMART: Pharmacist initiated management of antiretroviral treatment
PHC: Primary healthcare
NHI: National heath insurance
UHC: Universal health coverage
